# Comprehensive Bioinformatics Analysis of Toll-Like Receptors (TLRs) in Pan-Cancer

**DOI:** 10.1155/2022/4436646

**Published:** 2022-07-28

**Authors:** Wei Ping, Senyuan Hong, Yang Xun, Cong Li

**Affiliations:** ^1^Department of Thoracic Surgery, Tongji Hospital, Tongji Medical College, Huazhong University of Science and Technology, No. 1095 Jiefang Avenue, 430030 Wuhan, China; ^2^Department of Urology, Tongji Hospital, Tongji Medical College, Huazhong University of Science and Technology, No. 1095 Jiefang Avenue, 430030 Wuhan, China

## Abstract

**Background:**

To conduct a comprehensive bioinformatics analysis on the transcriptome signatures of Toll-like receptors (TLRs) in pan-cancer. *Materials and methods*. A total of 11,057 tissues consisting of 33 types of carcinoma in The Cancer Genome Atlas (TCGA) were retrieved, and then we further explored the correlation between TLRs' expression with tumorigenesis, immune infiltration, and drug sensitivity. We conducted a comprehensive bioinformatics analysis on TLR1 to 10 in pan-cancer, including differential expression analysis between normal and tumor tissues, differential immune subtype correlation, survival analysis, tumor immune infiltration estimating, stemness indices correlation, and drug responses correlation.

**Results:**

TLR2 was highly expressed in most types of tumors. TLR9 was hardly expressed compared to other TLR genes, which lead to TLR9 showing less correlation with both immune-estimate scores and stromal-estimate scores. All the TLRs were related with immune subtype of tumor samples that all of them were differentially expressed in differential immune subtype samples. The expression of TLRs was positively related with immune-estimate scores and stromal-estimate scores in almost all types of tumor. The expression of TLRs was negatively correlated with mRNA expression-based stemness scores (RNAss) in nearly almost type of tumors except kidney renal clear cell carcinoma (KIRC) and also negatively correlated with DNA methylation-based stemness scores (DNAss) in many types of tumors except adrenocortical carcinoma (ACC), cholangiocarcinoma (CHOL), KIRC, acute myeloid leukemia (LAML), low-grade glioma (LGG), testicular germ cell tumors (TGCT), thyroid carcinoma (THCA), thymoma (THYM), and uveal melanoma (UVM). The expression of TLR9 was significantly positively correlated with the drug sensitivity of fluphenazine, alectinib, carmustine, and 7−hydroxystaurosporine. TLR7 was significantly positively correlated with the drug sensitivity of alectinib.

**Conclusions:**

Our study reveals the significant role of TLRs family in pan-cancer and provides potential therapeutic strategies of cancer.

## 1. Introduction

Toll-like receptors (TLRs) are a family of transmembrane pattern recognition receptors that play essential roles in innate immunity for the detection of and defense against microbial pathogens [1]. TLRs are the first-line protective immune sentries that can recognize pathogen-associated molecular patterns (PAMPs), which typically include unmethylated double-stranded DNA (CpG), single-stranded RNA (ssRNA), lipoproteins, lipopolysaccharide (LPS), and flagellin [2]. They have been widely studied as the main mediators of innate immunity in animals, from insects to humans [3–5]. The discovery of TLRs as components that recognize the conserved structures in pathogens has greatly promoted the understanding of how the body perceives pathogen invasion, triggers innate immune responses, and initiates antigen-specific adaptive immunity [6].

It was reported that Drosophila strains with mutants of the Toll gene were highly susceptible to fungal infection, which was the first indication of the innate immune function of TLRs [7]. A human Toll homologue, now called TLR4, was then identified [8]. Currently, a total of 10 TLR family members have been identified in humans, and at least 13 have been discovered in mice. These are usually expressed by various immune cells, such as dendritic cells (DCs), macrophages, T-cell subsets, and B-cells. They are also expressed in nonimmune cells (e.g., epithelial cells and fibroblasts) in humans [9]. All TLRs include an N-terminal domain characterized by multiple leucine-rich repeats and a carboxyl-terminal TIR domain that interacts with TIR-containing adapters. Nucleic acid-sensing TLRs (TLR3, TLR7, TLR8, and TLR9) are located in the endoplasmic chamber, whereas the remaining TLRs are present on the plasma membrane [10, 11].

In recent years, TLRs have gained great interest in cancer research because of their role in tumor progression, and many therapeutic interventions for TLR have been developed or studied. Some studies have explored in detail the role of TLR regulation in cancer development [12–14]. Compared to that in normal patients, the expression of TLR1, 2, 4, and 8 mRNA was increased in patients with colorectal cancer [15]. TLRs have also been associated with prostate cancer, but they may be a double-edged sword in prostate tumorigenesis because they can both promote malignant transformation of epithelial cells thereby enhancing tumor growth and induce apoptosis, thus, inhibiting tumor progression [16]. In addition, the regulation of TLRs not only increases the susceptibility to infection from some microorganisms but also contributes to the development of cancer by altering the microbiota resulting in inflammation [17]. On one hand, TLRs play an essential role in tumor immunity by activating a variety of cells, such as DCs, T-cell subsets, and even tumor cells; on the other hand, the activation of TLRs can also lead to inflammation that results in tumor promotion [18].

However, the characteristics of TLRs differ, and different homologous types may have different effects on different tumor types. In addition, to date, no bioinformatics study has systematically investigated the transcriptional levels of each TLR across multiple cancers. Therefore, it is of great significance to study the expression patterns of TLRs in cancer tissues and to develop potential TLR-targeted drugs for treatment of tumors with differentially expressed TLRs. In this study, we analyzed the expression characteristics of TLR1 to TLR 10 in various cancer tissues using a variety of bioinformatics methods, comprehensively analyzed TLRs, and found that the transcriptional levels of TLRs were associated with stemness, tumor purity, and drug sensitivity in cancer tissues included in The Cancer Genome Atlas (TCGA).

## 2. Materials and Methods

### 2.1. Data Sources

The transcriptome profile, clinical phenotype information, survival information, immune subtype profile, and DNA and RNA stemness profiles of 33 types of tumors were downloaded from the Genomic Data Commons (GDC) TCGA sets or TCGA pan-cancer sets in the UCSC Xena database (http://xena.ucsc.edu/) on November 15, 2020. Transcriptome profiles containing both tumor and normal adjacent tumor (NAT) tissues yielded a total of 11,057 samples, coded as fragments per kilobase per million (FPKM).

### 2.2. Expression Status of TLRs across Multiple Cancer Types

We first extracted and visualized the pan-cancer expression of TLRs. We then selected the five most highly expressed TLRs for further differential expression analysis. We sorted the expression profiles for cancer types whose expression profiles retained the expression profile of NAT tissues, and they were BLCA, BRCA, CHOL, COAD, ESCA, GBM, HNSC, KICH, KIRC, KIRP, LIHC, LUAD, LUSC, PRAD, READ, STAD, THCA, and UCEC. We then extracted the expression of the 5 most highly expressed TLRs in these cancer types and performed differential expression analysis between tumors and NAT using the Wilcoxon test. In addition, for all the TLRs, we calculated the log2 fold change (logFC) of each TLR in these cancer types and presented it in a heatmap. Subsequently, we applied a correlation test to explore the coexpression of the 10 TLRs according to their expression profiles.

### 2.3. Prognostic Value of TLRs across Multiple Cancer Types

For each TLR gene and tumor type, we separately performed log-rank survival analysis (grouped by the medium expression of the TLR in each cancer type) and univariate Cox regression to explore the pan-cancer prognostic value of TLRs. We then visualized the survival curves with significant differences and drew a forest plot of the resulting hazard ratios (HRs) and their 95% confidence interval.

### 2.4. Immune Subtype Correlations, Stemness Indices Correlations, and Tumor Microenvironment (TME) Estimations

Based on the immune subtype profile of each TCGA sample downloaded from the UCSC Xena, we explored the differential expression status of TLRs in different immune subtypes using the Wilcoxon test. We further probed the correlation between the expression of TLRs and the stemness index of the tissue samples containing DNA methylation-based stemness scores (DNAss) and mRNA expression-based stemness scores (RNAss) across multiple cancer types using the Spearman's correlation test. In addition, we applied the ESTIMATE method to analyze the immune-estimate score and stromal-estimate score of each sample and then performed the Spearman's correlation test to examine the correlation between the expression of TLRs and these two scores.

### 2.5. Drug Sensitivity Analysis of TLRs across Multiple Cancer Types

Data including both expression of TLRs and drug sensitivity were retrieved from the CellMiner database ((https://discover.nci.nih.gov/cellminer/), which collects genomic and pharmacologic information for investigators to determine the correlation between gene expression and drug sensitivity in the NCI-60 cell line sets. Thus, we extracted the expression values of TLRs in NCI-60 cell lines and their corresponding drug sensitivities to different drugs and conducted a Pearson correlation test between the expression of TLRs and drug sensitivity to explore the drug sensitivity in patients.

### 2.6. TLRs in KIRC

Finally, as TLR expression performed well in predicting the overall survival for KIRC, we further explored the significance of TLRs in KIRC. We separately investigated the differential expression of TLRs among different immune subtypes, the correlation between TLR expression and stemness indices, and the correlation between TLR expression and ESTIMATE scores in KIRC samples. In addition, we explored the differential expression status of TLRs between stages I and IV to determine whether TLRs could serve as biomarkers of survival and progression in KIRC.

### 2.7. Statistical Analysis

All statistical analyses were conducted using the R software (version 4.0.2). Statistical significance was set at *p* < 0.05.

## 3. Results

### 3.1. Differential Expression Analysis of TLRs between Tumor and NAT Tissues

**Figure 1:**
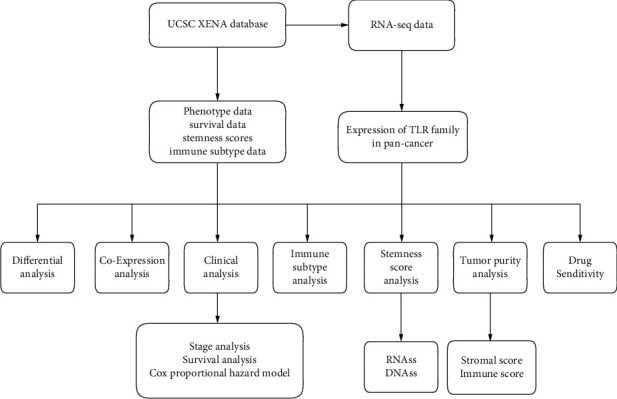
The study flow chart.

**Figure 2:**
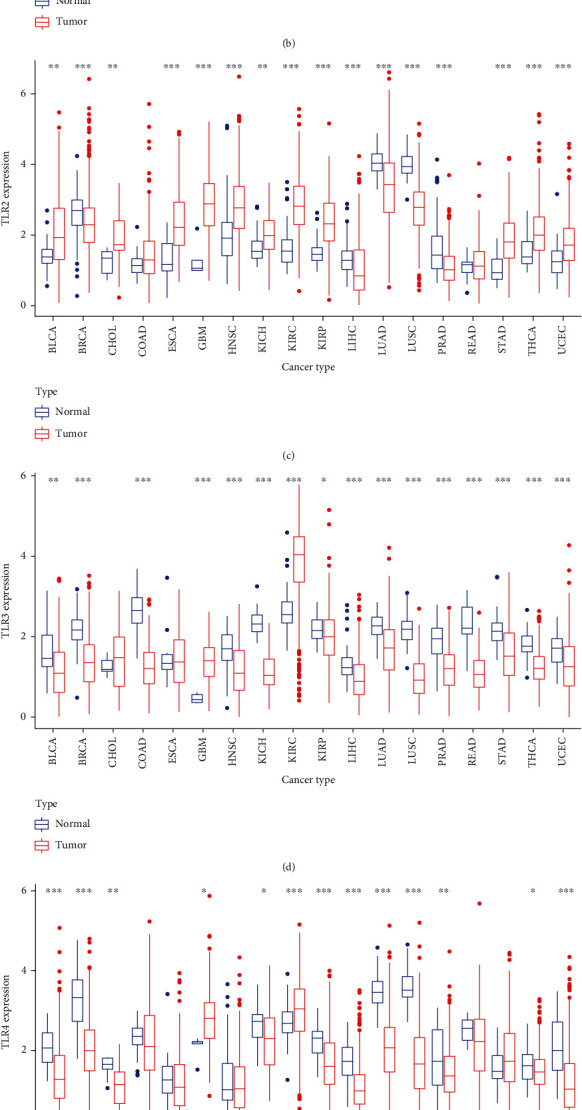
Expression status of TLRs. (a) Expression of TLRs in pan-cancer. (b) Differential expression of TLR1 in pan-cancer. (c) Differential expression of TLR2 in pan-cancer. (d) Differential expression of TLR3 in pan-cancer. (e) Differential expression of TLR4 in pan-cancer. (f) Differential expression of TLR5 in pan-cancer.

The flowchart of the study is summarized in Figure 1, and the abbreviations of the 33 tumor types in TCGA are provided in Table 1. The pan-cancer gene expression of TLR1 to TLR10 is displayed in Figure 2(a), and it seems that the expression of TLR9 was low compared to that of the other TLR genes. In addition, differential expression analysis with the Wilcoxon test was performed on the 10 TLR family genes between tumor and NAT tissues. Furthermore, the five most highly expressed genes, TLR1 to TLR5, were selected to show the differential expression status. TLR1 expression was significantly low in most types of cancers, except CHOL, GBM, and KIRC (Figure 2(b)). TLR2 was significantly expressed in most tumor types, except BRCA, LIHC, LUAD, LUSC, and PRAD (Figure 2(c)). TLR3 expression was significantly low in most type of tumors, except GBM and KIRC (Figure 2(d)). TLR4 expression was significantly low in most type of tumors, except GBM and KIRC (Figure 2(e)). TLR5 expression was significantly low in most type of tumors, except CHOL, GBM, and LIHC (Figure 2(f)).

### 3.2. Coexpression Analysis of TLRs across Multiple Cancer Types and Log-Rank Survival Analysis

**Figure 3:**
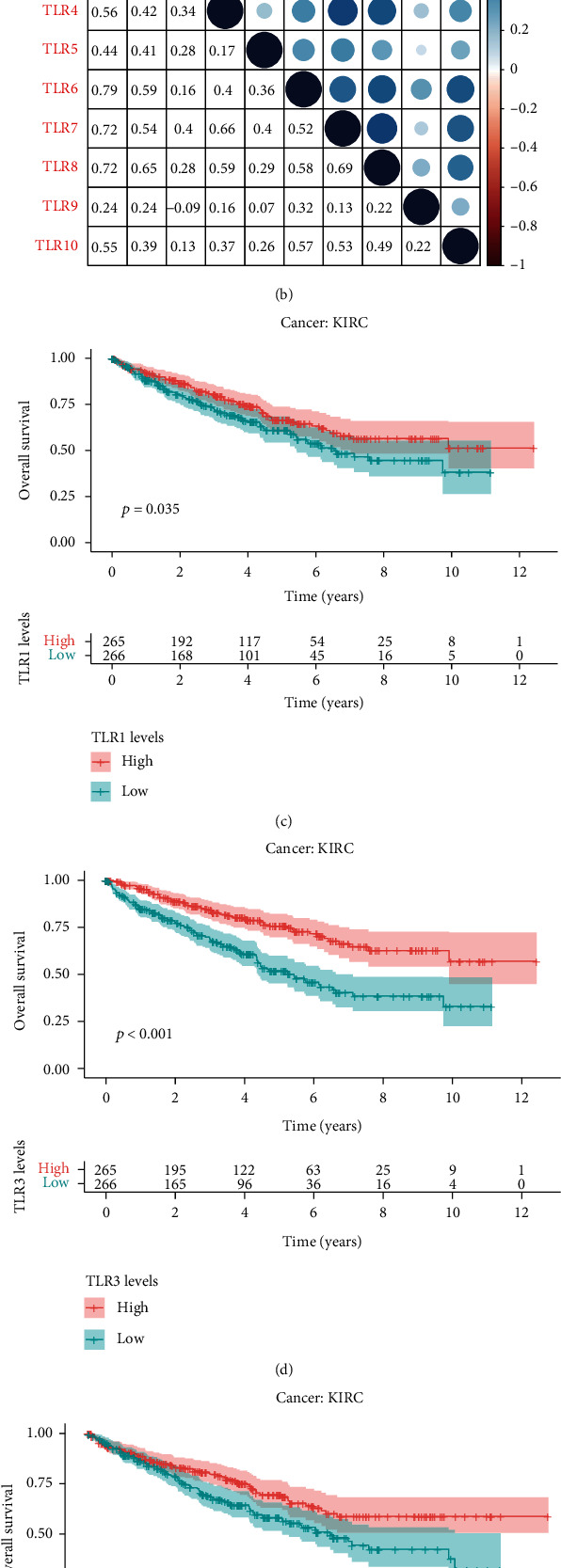
Coexpression of TLRs and survival curves in KIRC. (a) Differential expression status of TLR1 to TLR10 in pan-cancer. (b) Coexpression of TLRs in pan-cancer. (c) TLR1 as a candidate prognostic factor in KIRC. (d) TLR3 as a candidate prognostic factor in KIRC. (e) TLR4 as a candidate prognostic factor in KIRC. (f) TLR9 as a candidate prognostic factor in KIRC.

More detailed information about the differential expression status, including log2FC, is shown in Figure 3(a). It was obvious that TLR2 was highly expressed in most types of cancer, and TLR family members were least expressed in LUSC and LUAD. In addition, coexpression analysis of TLRs suggested that all TLRs were positively correlated with each other, except TLR3, which was negatively correlated with TLR9 (Figure 3(b)). We then employed Kaplan–Meier methods to plot survival curves and performed a log-rank analysis to investigate the prognostic value of TLRs for the 33 TCGA cancers. The prognostic values of TLRs with cancer type and *p* value are shown in Table 2. We then selected KIRC to plot the survival curves for the four TLR genes with prognostic values for KIRC, TLR1 (Figure 3(c)), TLR3 (Figure 3(d)), TLR4 (Figure 3(e)), and TLR9 (Figure 3(f)). Among these, low expression of TLR1, TLR3, and TLR4 was significantly associated with poor overall survival, while high expression of TLR9 was significantly associated with poor overall survival in KIRC.

### 3.3. Cox Regression and Immune Subtype Analysis

**Figure 4:**
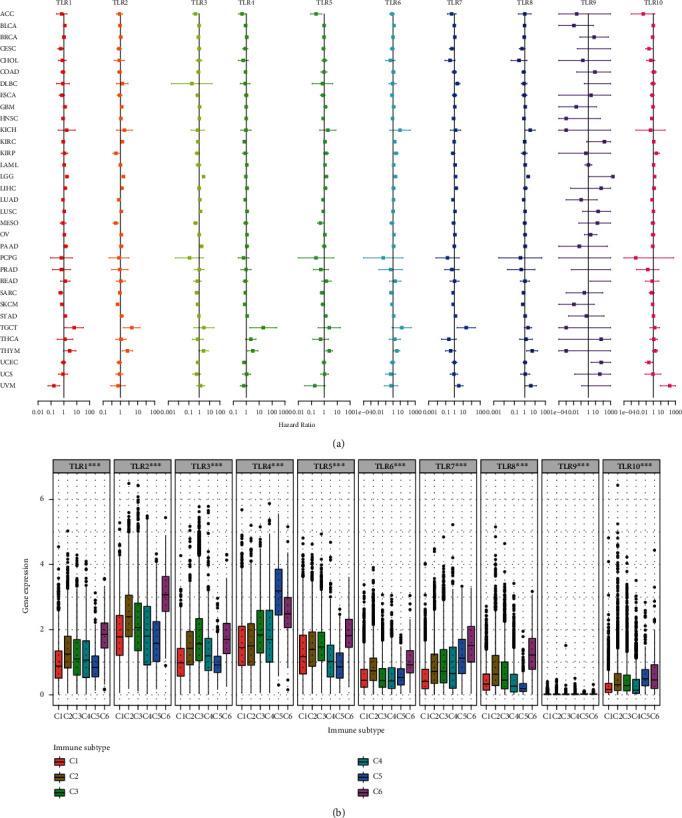
Cox regression and immune subtype analysis in pan-cancer. (a) Univariate Cox regression for each TLR gene in pan-cancer. (b) Differential expression of TLRs in differential immune subtype.

Univariate Cox proportional hazard regression was performed to explore the prognostic values of TLRs for the 33 types of cancer. Genes were considered a risk factor if the HR was >1 or a protective factor if the HR was <1. According to the forest plot (Figure 4(a)), we found that TLRs play a complex role in cancer prognosis, which is risky in some types of tumors but protective in the remaining types of tumors. In addition, we performed a Kruskal test on the expression of TLRs in the six immune subtypes across the 33 TCGA cancer types (Figure 4(b)). Interestingly, all TLRs were differentially expressed in the different immune subtype samples. Among them, TLR1, TLR2, TLR3, TLR5, TLR6, TLR7, and TLR8 showed the highest expression in C6 immune subtype samples, whereas TLR4 and TLR10 showed the highest expression in the C5 immune subtype.

### 3.4. TLRs and TME across Multiple Cancer Types

**Figure 5:**
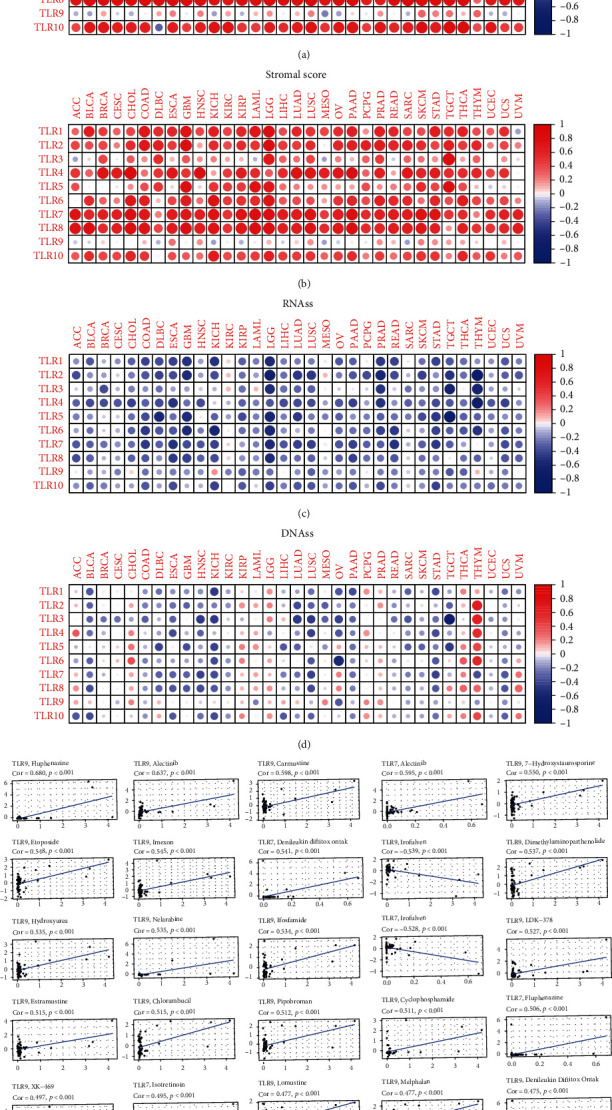
Stemness indices analysis, tumor microenvironment analysis, and drug sensitivity analysis in pan-cancer. (a) The correlation between immune score and expression of TLRs. (b) The correlation between stromal score and expression of TLRs. (c) The correlation between RNAss and expression of TLRs. (d) The correlation between DNAss and expression of TLRs. (e) Drug sensitivity of TLRs.

Immune-estimate scores and stromal-estimate scores of samples were calculated using the R package “ESTIMATE” [19], and Spearman's correlation test was used to explore the correlation between TLR expression and the TME. For the immune score, expression of TLRs was positively correlated with immune scores in almost all types of cancer, except TLR1 in UVM, TLR3, 4, and 5 in THYM, and TLR10 in DLBC (Figure 5(a)). In addition, for the stromal scores, the expression of TLRs was positively correlated with stromal scores in almost all types of cancer, except TLR1 in UVM and TLR3 in ACC, LAML, MESO, and READ (Figure 5(b)). TLR9 showed low correlation with both immune and stromal scores, which may be due to the low expression of TLR9 in all the tumor samples.

### 3.5. TLRs and Stemness Indices across Multiple Cancer Types

We downloaded the stemness indices for all the samples from the UCSC Xena database, which were calculated using the one-class logistic regression (OCLR) as proposed by Malta et al. [20]. Two types of stemness indices were assessed: DNAss and RNAss. Interestingly, the expression of TLRs was negatively correlated with RNAss in nearly all types of cancer, except KIRC (Figure 5(c)), and negatively correlated with DNAss in many types of cancer, except ACC, CHOL, KIRC, LAML, LGG, TGCT, THCA, THYM, and UVM (Figure 5(d)). Among the DNAss scores, nearly all TLRs, except for TLR7 and TLR9, were positively correlated with DNAss in THYM samples.

### 3.6. TLRs and Drug Responses across Multiple Cancer Types

The expression profile of NCI-60 cancer cell lines and their drug sensitivity were downloaded from the CellMiner database; the Pearson correlation test was then performed to further analyze the correlation between the expression and the response to 263 antineoplastic drugs. All results with significant correlation between TLRs and drug sensitivity are displayed in Supplementary Table (available here), and the 25 most significant results with the smallest *p* value are shown as scatter plots ranked by *p* value (Figure 5(e)). Among them, the five most significant correlations were as follows: the expression of TLR9 had a significant positive correlation with the response to fluphenazine (coefficient = 0.680, *p* < 0.001), alectinib (coefficient = 0.637, *p* < 0.001), carmustine (coefficient = 0.598, *p* < 0.001), and 7−hydroxystaurosporine (coefficient = 0.550, *p* < 0.001), while TLR7 had a significant positive correlation with alectinib (coefficient = 0.595, *p* < 0.001).

### 3.7. TLRs in KIRC

**Figure 6:**
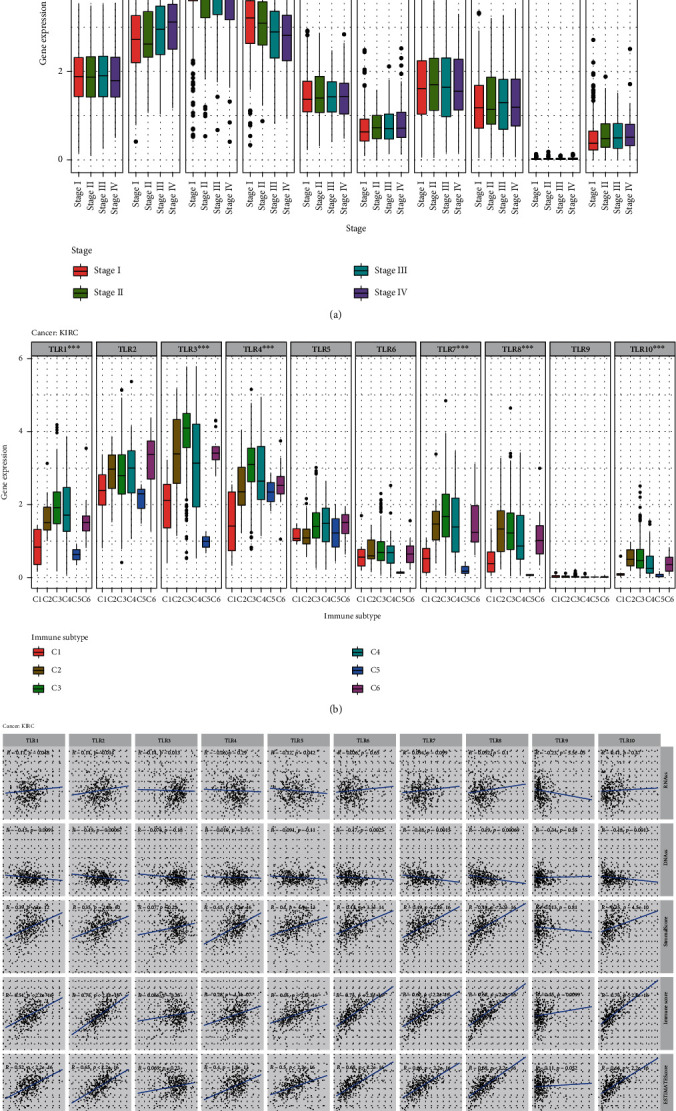
TLRs in KIRC. (a) Differential expression of TLRs between stage I and stage IV in KIRC. (b) Differential expression of TLRs in different immune subtype in KIRC. (c) Correlation between the expression of TLRs and stemness indices, tumor microenvironment.

Finally, we explored TLRs in KIRC by comparing the transcriptional expression of TLRs at different stages of KIRC, comparing the differential expression of TLRs in different immune subtypes, and investigating the correlation between TLRs and stemness indices or tumor purity in KIRC. TLR2, TLR3, TLR4, and TLR10 were significantly differentially expressed between stages I and IV (*p* < 0.05) (Figure 6(a)), and TLR1, TLR3, TLR4, TLR7, TLR8, and TLR10 were significantly differentially expressed between the C1 and C6 immune subtypes (*p* < 0.001) (Figure 6(b)). For RNAss in the KIRC samples, TLR5 and TLR9 had significant negative correlations (correlation coefficient = −0.12, *p* = 0.042 and correlation coefficient = −0.23, *p* < 0.001, respectively), but TLR1, TLR2, and TLR3 had significant positive correlations (correlation coefficient = 0.11, *p* = 0.048; correlation coefficient = 0.14, *p* = 0.014; and correlation coefficient = 0.14; *p* = 0.013, respectively). For DNAss in the KIRC samples, it was interesting that all the TLRs were negatively correlated in KIRC patients, among which TLR1, TLR2, TLR6, TLR7, TLR8, and TLR10 were significant at *p* < 0.05. In addition, all the TLRs had significant positive correlations with the immune scores, stromal scores, and ESTIMATE scores. Among them, TLR1, TLR2, TLR4, TLR5, TLR6, TLR7, TLR8, and TLR10 were positively correlated with stromal scores (*p* < 0.05); TLR1, TLR2, TLR4, TLR5, TLR6, TLR7, TLR8, TLR9, and TLR10 were positively correlated with immune scores (*p* < 0.05); and TLR1, TLR2, TLR4, TLR5, TLR6, TLR7, TLR8, and TLR10 were positively correlated with the ESTIMATE scores (*p* < 0.05) (Figure 6(c)).

## 4. Discussion

Many studies have demonstrated that several cellular and molecular mechanisms can help tumors escape the body's natural immune response [21, 22]. The importance of immune regulation in cancer progression can be explained by the increase in the number of immunosuppressive factors and cells and the lack of immune system-activating signals in the TME. TLRs are important receptors that activate immune cells and have been reported to play an important role in cancers, such as bladder cancer and colorectal cancer [23, 24]. This makes TLRs suitable targets for ligand drug discovery strategies to establish new therapeutics for cancer [25]. Hence, it is worthwhile to explore the role of TLRs in tumor development. TLRs can upregulate the expression of costimulatory molecules, such as CD40, CD80, and CD86, and cytokines, such as IL-12, thus stimulating other immune cells, including T lymphocytes [26, 27]. However, TLR expression can lead to tumor growth by stimulating other cells, including cancer cells [28].

In this study, we explored the relationship between TLR transcriptional expression and TCGA tumor characteristics, including the TME, clinical significance, immune subtypes, stem cells, and drug response. We found that TLR isotypes have a significant effect on tumorigenesis. First, we analyzed the differential expression of 33 TCGA cancer types in 11,057 samples (including 10,327 tumor samples and 730 paracancerous samples). Through multidimensional analysis, we found significant differences in TLR expression levels among different cancer types. Survival and Cox proportional hazard regression analyses were also performed. For some types of cancers, we found a statistically significant difference in survival between patients with high and low TLR expression, suggesting that TLRs may be a potential prognostic indicator for clinical applications. Furthermore, we performed drug response analysis to explore the relationship between drug sensitivity and TLRs. This is expected to provide insights for new cancer therapies.

In our study, TLR2 was highly expressed in most cancer types. This result is similar to that of most previous studies [29–31]. Gergen et al. [32] reported that TLR2 activation induces the proliferation of lung adenocarcinoma cells by activating NF-*ĸ*B. As a special link between lung cancer cells and mesenchymal stem cells in the TME, TLR2 promotes crosstalk and ultimately promotes changes in the tumor-supporting phenotype of mesenchymal cells [33]. Furthermore, the expression of TLR2 protein was shown to be upregulated in colon cancer and significantly correlated with a low overall survival rate of patients with colon cancer [34, 35]. Thus, the TLR2 signaling pathway may be an important potential therapeutic target in cancer.

In our study, we found that TLR9 was hardly expressed compared to the other TLR genes, which led to TLR9 showing less correlation with both immune and stromal scores. However, several studies have reported that TLR9 is associated with the development of cancers, especially gynecologic cancer [36, 37]. The activation of TLR9 on DCs and plasmacytoid DCs promotes the secretion of a large amount of type I IFN, which has both direct (tumor cell inhibitory effect) and indirect (antitumor immune responses) effects on cancer cells and is most evident in the early stages of antitumor immune responses [38].

Thorsson et al. [39] identified the immune landscape of cancer in the C1-C6 immune subtypes. In our study, we classified tumor samples by representative immune signatures and detected the RNA-seq levels of TLR 1-10 in C1 to C6. Interestingly, all TLRs were differentially expressed in different immune subtype samples. The TME, including the extracellular matrix, tumor vascular system, and tumor cell types, is closely related to immune functions and has an important impact on treatment response and clinical prognosis [40]. TLRs are expressed in the TME [41]. We further confirmed this information by extracting data on the fractions of stromal and immune cells in tumor samples from the 33 TCGA cancer types by calculating stromal scores, immune scores, and ESTIMATE scores. TLR expression was positively correlated with immune and stromal scores in almost all cancer types. On one hand, TLRs are expressed during programmed cell death induced by TME; on the other hand, they trigger the release of cytokines and chemokines in the TME and recruit immune cells to further release proinflammatory cytokines, angiogenic factors, and growth factors, such as TGF *β*, IL-8, CXCR4, ICAM-1, and VEGF. TLRs can repair the antitumor function and apoptotic response of antigen-presenting cells and effector T-cells [42, 43]. TLR signaling pathways play an essential role in controlling tumor progression, metastasis, recurrence, and chemotherapy tolerance through inappropriate immune enhancement and antitumor immunity [44].

Stemness was used to distinguish the stem cell-like characteristics of the tumor, such as self-renewal and dedifferentiation [45]. Two types of stemness indices were assessed: DNAss and RNAss [46]. We found that the expression of TLRs was negatively correlated with RNAss in nearly all types of cancers, except KIRC, and negatively correlated with DNAss in many types of cancers, except ACC, CHOL, KIRC, LAML, LGG, TGCT, THCA, THYM, and UVM. TLR3 activation facilitates the expression of stemness-associated genes, including OCT3/4, NANOG, and SOX2 [47]. TLR4 expression in HCC is associated with increased stem-like properties [48]. NF-𝜅B, activated by TLR signaling, was closely aligned with proliferation, invasion, and tumorigenesis [49].

Our study also found that the transcriptional expression levels of TLR7 and TLR9 were associated with drug response. Among them, the expression of TLR9 had a significant positive correlation with drug sensitivity to fluphenazine, alectinib, carmustine, and 7−hydroxystaurosporine. There was a significant positive correlation between TLR7 and the drug sensitivity of alectinib. These results have clinical relevance for guiding selection of antitumor therapies.

Finally, we explored the relationship between TLRs and KIRC. TLR2, TLR3, TLR4, and TLR10 were significantly differentially expressed between stages I and IV. TLR1, TLR3, TLR4, TLR7, TLR8, and TLR10 were significantly differentially expressed between C1 and C6 immune subtypes. All TLRs were positively correlated with immune, stromal, and ESTIMATE scores. Morikawa et al. [50] reported that TLR3 was overexpressed in KIRC, suggesting that the TLR3 pathway may be a novel therapeutic target in KIRC. Moreover, the expression of TLR9 is an independent prognostic marker of KIRC, and the loss of TLR9 expression is related to poor prognosis of KIRC [51]. Our results provide guidance for further exploration of the role of TLRs in KIRC.

Although this is the first study to multidimensionally analyze TLRs across multiple cancer types, it has some limitations. First, our results have not been verified using other independent databases; thus, it is necessary to validate the conclusions by generating our own data and using other public databases in the future. Second, this was a dry lab study [52], and we have not explored the underlying mechanisms behind the bioinformatics analyses through molecular and animal experiments. Finally, we studied the relationship between TLR family members and various combinatorial data. However, biometric correlations may not clarify the mechanisms of interaction and regulation directly; thus, further studies are needed to verify these potential mechanisms via laboratory-based molecular experiments. Further investigation is needed to determine the potential of TLRs and their coactivators as therapeutic targets in cancer.

## 5. Conclusions

TLRs were expressed differently in different cancer types and different immune subtype tissue and were positively correlated with immune-estimate scores and stromal-estimate scores. The expression of TLR9 had a significant positive correlation with the drug sensitivities to fluphenazine, alectinib, carmustine, and 7−hydroxystaurosporine. TLR7 had a significant positive correlation with alectinib sensitivity. We demonstrated the significant pan-cancer role of the TLR family and potential therapeutic strategies for cancer. However, further laboratory studies are required to confirm our results.

## Figures and Tables

**Table 1:** Abbreviations of the 33 tumor types in TCGA.

Abbreviation	Tumor type
ACC	Adrenocortical carcinoma
BLCA	Bladder urothelial carcinoma
BRCA	Breast invasive carcinoma
CESC	Cervical squamous cell carcinoma and endocervical adenocarcinoma
CHOL	Cholangiocarcinoma
COAD	Colon adenocarcinoma
DLBC	Lymphoid neoplasm diffuse large B-cell lymphoma
ESCA	Esophageal carcinoma
GBM	Glioblastoma multiforme
HNSC	Head and neck squamous cell carcinoma
KICH	Kidney chromophobe
KIRC	Kidney renal clear cell carcinoma
KIRP	Kidney renal papillary cell carcinoma
LAML	Acute myeloid leukemia
LGG	Brain lower grade glioma
LIHC	Liver hepatocellular carcinoma
LUAD	Lung adenocarcinoma
LUSC	Lung squamous cell carcinoma
MESO	Mesothelioma
OV	Ovarian serous cystadenocarcinoma
PAAD	Pancreatic adenocarcinoma
PCPG	Pheochromocytoma and paraganglioma
PRAD	Prostate adenocarcinoma
READ	Rectum adenocarcinoma
SARC	Sarcoma
SKCM	Skin cutaneous melanoma
STAD	Stomach adenocarcinoma
TGCT	Testicular germ cell tumors
THCA	Thyroid carcinoma
THYM	Thymoma
UCEC	Uterine corpus endometrial carcinoma
UCS	Uterine carcinosarcoma
UVM	Uveal melanoma

**Table 2:** Detailed information of the survival analysis of the TLRs family in pan-cancer with significant *p* value.

Gene	Cancer type	*p* value
TLR1	KIRC	0.034688969
TLR1	LGG	0.000296933
TLR1	SARC	0.026721888
TLR1	SKCM	0.000697683
TLR1	UVM	0.002732005
TLR2	LGG	1.65E-05
TLR2	LUAD	0.008433019
TLR2	MESO	0.017243009
TLR2	SKCM	2.17E-06
TLR2	TGCT	0.018921076
TLR2	THYM	0.009423895
TLR3	KIRC	2.94E-07
TLR3	KIRP	0.004130991
TLR3	LGG	0.000104245
TLR3	MESO	0.002692585
TLR3	PAAD	0.024365165
TLR3	SARC	0.009139017
TLR3	SKCM	0.000167396
TLR3	TGCT	0.042432124
TLR3	UCEC	0.031991718
TLR4	ACC	0.007177616
TLR4	KIRC	0.007164204
TLR4	LAML	0.044171562
TLR4	LUAD	0.028500171
TLR4	SKCM	7.46E-05
TLR4	TGCT	0.021867869
TLR4	THYM	0.020130819
TLR4	UCEC	0.00545563
TLR5	ACC	0.01419818
TLR5	ESCA	0.040248748
TLR5	LGG	0.014075805
TLR5	OV	0.036628212
TLR5	SKCM	0.022197327
TLR5	STAD	0.008021708
TLR5	THYM	0.005545537
TLR6	BLCA	0.036456726
TLR6	ESCA	0.01912187
TLR6	KIRP	0.008085447
TLR6	LGG	0.003399361
TLR6	SKCM	0.003736075
TLR7	DLBC	0.032371891
TLR7	LAML	0.01921935
TLR7	LGG	0.00593611
TLR7	LUAD	0.000486804
TLR7	SARC	0.016297397
TLR7	SKCM	0.001124964
TLR7	UVM	0.03400155
TLR8	LAML	0.032090719
TLR8	LGG	0.003214408
TLR8	SKCM	1.26E-06
TLR8	THYM	0.020533224
TLR8	UVM	0.004248326
TLR9	KIRC	0.020215421
TLR9	LAML	0.038214202
TLR9	UCEC	2.97E-05
TLR10	CESC	0.02579765
TLR10	COAD	0.041270017
TLR10	HNSC	0.013737092
TLR10	LGG	0.003769908
TLR10	LUAD	0.000369848
TLR10	READ	0.028148853
TLR10	SARC	0.000659968
TLR10	SKCM	1.31E-05
TLR10	UCEC	0.003987076

## Data Availability

Source data of this study were derived from the public repositories, as indicated in the section of “Materials and Methods” of the manuscript. And all data that support the findings of this study are available from the corresponding author upon reasonable request.

## Abbreviations

TLRs: Toll-like receptors
RNAss: mRNA expression-based stemness scores
KIRC: Kidney renal clear cell carcinoma
DNAss: DNA methylation-based stemness scores
ACC: Adrenocortical carcinoma
CHOL: Cholangiocarcinoma
LAML: Acute myeloid leukemia
LGG: Low-grade glioma
TGCT: Testicular germ cell tumors
THCA: Thyroid carcinoma
THYM: Thymoma
UVM: Uveal melanoma
PAMPs: Pathogen-associated molecular patterns
ssRNA: Single-stranded RNA
LPS: Lipopolysaccharide
DCs: Dendritic cells
TCGA: The Cancer Genome Atlas
HR: Hazard ration
OCLR: One-class logistic regression.

## Supplementary Materials

Supplementary Materials Supplementary Table. Detailed information about the drug sensitivity of TLRs.13.
