# Supplementary material for: Comprehensive Bioinformatics Analysis of Toll-Like Receptors (TLRs) in Pan-Cancer
Source: Biomed Res Int. 2022 Jul 28;2022:4436646. doi: 10.1155/2022/4436646 (PMC9352480; doi:10.1155/2022/4436646)
Supplement: Supplementary Materials — Supplementary Table. Detailed information about the drug sensitivity of TLRs.13. [file 4436646.f1.pdf]

Suplementary Table. Detailed information about the drug sensitivity of TLRs.

Gene	Drug	cor	pvalue
TLR9	Fluphenazine	0.680496	2.21E-09
TLR9	Alectinib	0.637334	4.36E-08
TLR9	Carmustine	0.59831	4.45E-07
TLR7	Alectinib	0.594836	5.39E-07
TLR9	7-Hydroxystaurosporine	0.549878	5.34E-06
TLR9	Etoposide	0.547573	5.96E-06
TLR9	Imexon	0.545371	6.61E-06
TLR7	Denileukin Diftitox Ontak	0.540666	8.22E-06
TLR9	Irofulven	-0.5392	8.79E-06
TLR9	Dimethylaminoparthenolide	0.536639	9.87E-06
TLR9	Hydroxyurea	0.535276	1.05E-05
TLR9	Nelarabine	0.534835	1.07E-05
TLR9	Ifosfamide	0.534254	1.10E-05
TLR7	Irofulven	-0.52814	1.44E-05
TLR9	LDK-378	0.527037	1.52E-05
TLR9	Estramustine	0.514739	2.58E-05
TLR9	Chlorambucil	0.514505	2.61E-05
TLR9	Pipobroman	0.511984	2.90E-05
TLR9	Cyclophosphamide	0.510963	3.02E-05
TLR7	Fluphenazine	0.505542	3.79E-05
TLR9	XK-469	0.496931	5.37E-05
TLR7	Isotretinoin	0.495354	5.72E-05
TLR9	Lomustine	0.477229	0.000115556
TLR9	Melphalan	0.476729	0.000117753
TLR9	Denileukin Diftitox Ontak	0.47538	0.000123872
TLR3	Tyrothricin	-0.46478	0.000183134
TLR5	Olaparib	0.462569	0.000198352
TLR7	LDK-378	0.461385	0.000206978
TLR9	Nitrogen mustard	0.460885	0.000210719
TLR9	Teniposide	0.457912	0.0002343
TLR9	Arsenic trioxide	0.457805	0.000235193
TLR9	LMP-400	0.456865	0.000243165
TLR9	Oxaliplatin	0.449765	0.000311806
TLR9	Uracil mustard	0.449308	0.000316776
TLR9	Asparaginase	0.448462	0.000326175
TLR9	Chelerythrine	0.446291	0.000351461
TLR7	Imiquimod	0.44293	0.000394129
TLR9	Entinostat	0.442212	0.000403846
TLR7	Megestrol acetate	0.433224	0.000545172
TLR9	PX-316	0.428454	0.00063715
TLR9	Valrubicin	0.423695	0.000742722
TLR7	Entinostat	0.42301	0.000759159
TLR1	Denileukin Diftitox Ontak	0.421963	0.000784918
TLR9	Dromostanolone Propionate	0.421355	0.000800236
TLR7	Nelfinavir	0.418076	0.000887561
TLR9	Triethylenemelamine	0.415488	0.000962503
TLR9	Thiotepa	0.415069	0.000975134
TLR7	Estramustine	0.41378	0.001015024
TLR3	Alvespimycin	-0.4123	0.001062751
TLR5	Ixazomib citrate	-0.41177	0.001080262
TLR9	Parthenolide	0.410425	0.001125763
TLR2	Allopurinol	0.40535	0.001314012
TLR9	Raloxifene	0.399475	0.001566914
TLR9	Bendamustine	0.398053	0.001634338
TLR9	Epirubicin	0.396218	0.001725149
TLR9	Raltitrexed	0.392872	0.00190252

TLR2	ABT-199	0.390289	0.002050378
TLR5	Precursor Intermediate to TDP 6657	0.385202	0.002372001
TLR9	Dexamethasone Decadron	0.381996	0.00259704
TLR9	Fenretinide	0.380815	0.002684616
TLR9	Crizotinib	0.379013	0.002823321
TLR9	Isotretinoin	0.378539	0.002860818
TLR5	Pralatrexate	-0.3784	0.002872304
TLR9	Ethinyl estradiol	0.376113	0.003059973
TLR2	Vinorelbine	-0.37428	0.003218563
TLR9	Belinostat	0.369839	0.00363304
TLR9	AP-26113	0.369582	0.003658405
TLR4	Megestrol acetate	0.368833	0.00373326
TLR7	Elesclomol	0.367962	0.003821994
TLR3	Chelerythrine	-0.36481	0.004159264
TLR9	Calusterone	0.362582	0.004412711
TLR3	Idelalisib	0.362436	0.004429786
TLR9	Palbociclib	0.361447	0.004547231
TLR7	AP-26113	0.360816	0.004623553
TLR7	7-Hydroxystaurosporine	0.360659	0.004642648
TLR5	BML-277	-0.35842	0.004923183
TLR7	Dimethylaminoparthenolide	0.358401	0.004926102
TLR9	kahalide f	-0.35837	0.004930029
TLR9	Daunorubicin	0.357324	0.005066519
TLR7	Celecoxib	0.35704	0.005104155
TLR7	Dromostanolone Propionate	0.355933	0.005253123
TLR10	Elesclomol	0.355594	0.005299538
TLR1	Megestrol acetate	0.351035	0.005959208
TLR9	Perifosine	0.350355	0.006063591
TLR9	BN-2629	0.346841	0.006628691
TLR6	Trametinib	0.346163	0.006743031
TLR7	Imexon	0.344451	0.007039061
TLR1	Entinostat	0.341978	0.007486725
TLR9	6-Mercaptopurine	0.34188	0.007504861
TLR9	Vorinostat	0.340409	0.007783492
TLR7	Oxaliplatin	0.340021	0.007858509
TLR3	Lapachone	-0.33918	0.008022417
TLR2	Elliptinium Acetate	-0.33832	0.008195121
TLR7	Ethinyl estradiol	0.337846	0.008290345
TLR9	Sonidegib	-0.33735	0.008391349
TLR1	Dasatinib	-0.33648	0.00857253
TLR9	Fostamatinib	0.334964	0.008894472
TLR5	Oxaliplatin	-0.33424	0.009052245
TLR9	Carboplatin	0.33119	0.009742983
TLR2	Vinblastine	-0.33081	0.00983269
TLR1	XL-147	0.330646	0.009870915
TLR10	Imiquimod	0.330327	0.009946741
TLR7	Tegafur	0.330024	0.01001913
TLR5	AT-13387	-0.32943	0.010163012
TLR7	Etoposide	0.327935	0.010530533
TLR9	Dacarbazine	0.323203	0.011773564
TLR5	Methotrexate	-0.32275	0.011898278
TLR9	Irinotecan	0.322187	0.012056504
TLR7	Carmustine	0.322037	0.01209871
TLR1	Estramustine	0.321226	0.012329305
TLR5	Nilotinib	-0.31958	0.012809705
TLR5	3-Bromopyruvate (acid)	-0.31836	0.013173716
TLR9	Dexrazoxane	0.318223	0.013216752
TLR9	RH1	0.315881	0.013946487

TLR9	3-Bromopyruvate (acid)	0.315431	0.01409051
TLR1	Acetalax	-0.31534	0.014118756
TLR9	Nelfinavir	0.314338	0.014445923
TLR1	Pelitrexol	-0.3139	0.014589431
TLR5	Tanespimycin	-0.3138	0.014624186
TLR9	Trametinib	-0.31146	0.015419833
TLR9	Decitabine	0.310392	0.015793336
TLR1	Ethinyl estradiol	0.310288	0.015830446
TLR5	Dabrafenib	-0.31019	0.015863835
TLR5	Procarbazine	0.310093	0.015899794
TLR4	Isotretinoin	0.307516	0.016842443
TLR9	Batracylin	0.30545	0.017631918
TLR9	Pipamperone	0.304986	0.017813756
TLR2	Paclitaxel	-0.304	0.018206181
TLR7	Belinostat	0.303964	0.018219409
TLR4	Entinostat	0.303094	0.018570974
TLR9	Cisplatin	0.301788	0.019109415
TLR9	Celecoxib	0.301227	0.019344718
TLR10	Paclitaxel	0.299407	0.020125325
TLR6	Cobimetinib (isomer 1)	0.298626	0.020468501
TLR5	Vincristine	-0.29819	0.020663993
TLR5	Acetalax	0.296638	0.021364469
TLR4	AT-13387	-0.29556	0.0218647
TLR9	Fludarabine	0.295055	0.022101646
TLR7	Epirubicin	0.292304	0.023433471
TLR2	Actinomycin D	-0.29178	0.023694314
TLR5	Triciribine phosphate	0.289795	0.024706953
TLR10	Entinostat	0.289262	0.024984541
TLR5	isacodyl, active ingredient of viraple	0.288047	0.025627986
TLR5	Vandetanib	0.286696	0.026359545
TLR3	Cabozantinib	0.28583	0.026837894
TLR9	Megestrol acetate	0.285436	0.027057923
TLR9	Eribulin mesilate	0.285418	0.027068106
TLR5	Itraconazole	0.284816	0.027406991
TLR4	Lapatinib	-0.28458	0.027541793
TLR9	Tamoxifen	0.282904	0.028507907
TLR2	Acrichine	-0.2829	0.028510138
TLR6	Chelerythrine	-0.28176	0.029185063
TLR3	geldanamycin analog	-0.2816	0.029279939
TLR9	Doxorubicin	0.280755	0.029789383
TLR9	Triapine	0.280398	0.030006823
TLR8	Paclitaxel	0.279363	0.030644879
TLR9	Cytarabine	0.279085	0.030817987
TLR9	Mitoxantrone	0.278547	0.031155429
TLR9	Dasatinib	-0.27788	0.031578947
TLR9	Tegafur	0.276507	0.032464498
TLR3	O-6-Benzylguanine	0.275985	0.032806358
TLR6	Acetalax	-0.27595	0.032831005
TLR1	3-Bromopyruvate (acid)	-0.2757	0.032993082
TLR4	Acetalax	-0.27476	0.033621244
TLR10	Azacitidine	0.274709	0.033655248
TLR1	isacodyl, active ingredient of viraple	-0.27429	0.033941088
TLR2	BML-277	0.274058	0.034095077
TLR9	Imiquimod	0.272823	0.034943794
TLR2	Epirubicin	-0.27119	0.036094706
TLR7	Valrubicin	0.270402	0.036656793
TLR7	Teniposide	0.269452	0.03734849
TLR5	Gefitinib	0.268831	0.037805617

TLR6	XL-147	0.268328	0.038179728
TLR6	Gefitinib	0.266974	0.039201889
TLR6	3-Bromopyruvate (acid)	-0.26676	0.039364857
TLR9	Idarubicin	0.265841	0.040074359
TLR1	Isotretinoin	0.265743	0.040150461
TLR5	RH1	-0.26483	0.040864715
TLR4	By-Product of CUDC-305	-0.2647	0.040966634
TLR1	ABT-199	0.264652	0.041007554
TLR6	Midostaurin	-0.26406	0.041479682
TLR6	Entinostat	0.263522	0.041910237
TLR4	okadaic acid	0.262281	0.042921128
TLR1	Selumetinib	0.261341	0.043699796
TLR8	Elesclomol	0.261175	0.04383875
TLR1	okadaic acid	0.260784	0.044167008
TLR4	Ibrutinib	-0.26053	0.044383554
TLR5	Lapachone	-0.2601	0.044744843
TLR7	Ifosfamide	0.259879	0.044934312
TLR10	Tegafur	0.259877	0.044936164
TLR7	Eribulin mesilate	0.258361	0.046246627
TLR3	Ibrutinib	0.258267	0.046328457
TLR2	Staurosporine	0.257788	0.046749207
TLR3	Axitinib	0.257271	0.047207891
TLR1	Cobimetinib (isomer 1)	0.256934	0.047508414
TLR2	Tyrothricin	-0.25689	0.04754907
TLR8	Benzimate	0.256822	0.047608351
TLR2	Calusterone	0.256746	0.047676271
TLR2	Eribulin mesilate	-0.25639	0.047997908
TLR6	Fenretinide	-0.25625	0.048122288
TLR1	Triciribine phosphate	-0.25549	0.048812041
TLR7	Salinomycin	0.254519	0.049707352
